# Global estimation of areas with suitable environmental conditions for mariculture species

**DOI:** 10.1371/journal.pone.0191086

**Published:** 2018-01-19

**Authors:** Muhammed A. Oyinlola, Gabriel Reygondeau, Colette C. C. Wabnitz, Max Troell, William W. L. Cheung

**Affiliations:** 1 Nippon Foundation-Nereus Program and Changing Ocean Research Unit, Institute for the Oceans and Fisheries, The University of British Columbia, Vancouver, Canada; 2 Stockholm Resilience Centre, Stockholm University, Stockholm, Sweden; 3 The Beijer Institute, The Swedish Royal Academy of Sciences, Stockholm, Sweden; Universita degli Studi di Napoli Federico II, ITALY

## Abstract

Aquaculture has grown rapidly over the last three decades expanding at an average annual growth rate of 5.8% (2005–2014), down from 8.8% achieved between 1980 and 2010. The sector now produces 44% of total food fish production. Increasing demand and consumption from a growing global population are driving further expansion of both inland and marine aquaculture (i.e., mariculture, including marine species farmed on land). However, the growth of mariculture is dependent on the availability of suitable farming areas for new facilities, particularly for open farming practices that rely on the natural oceanic environmental parameters such as temperature, oxygen, chlorophyll etc. In this study, we estimated the marine areas within the exclusive economic zones of all countries that were suitable for potential open ocean mariculture activities. To this end, we quantify the environmental niche and inferred the global habitat suitability index (HSI) of the 102 most farmed marine species using four species distribution models. The average weighted HSI across the four models suggests that 72,000,000 km^2^ of ocean are to be environmentally suitable to farm one or more species. About 92% of the predicted area (66,000,000 km^2^) is environmentally suitable for farming finfish, 43% (31,000,000 km^2^) for molluscs and 54% (39,000,000 km^2^) for crustaceans. These predictions do not consider technological feasibility that can limit crustaceans farming in open waters. Suitable mariculture areas along the Atlantic coast of South America and West Africa appear to be most under-utilized for farming. Our results suggest that factors other than environmental considerations such as the lack of socio-economic and technological capacity, as well as aqua feed supply are currently limiting the potential for mariculture expansion in many areas.

## Introduction

**Fig 1:**
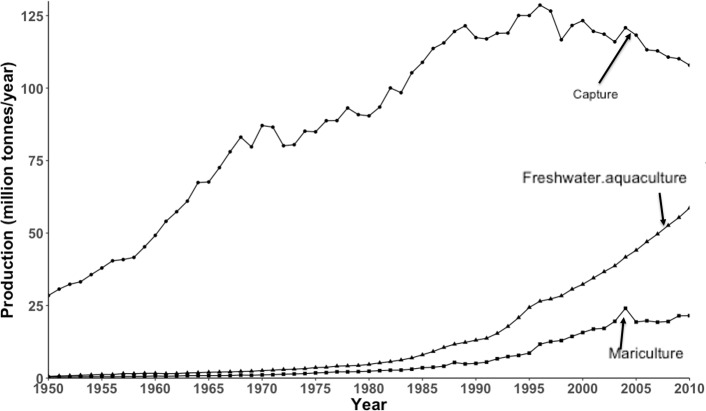
Global trends in food fish production from 1950 to 2010 with production from capture fisheries, freshwater aquaculture and mariculture. Data from Sea Around Us (2010) and FAO (2012).

Fish and other aquatic organisms, particularly from the marine environment, significantly contribute to the nutritional security and well-being of human society. Many coastal communities in tropical developing countries are highly dependent on fish as an important source of nutrients [1–3]. Given projected increases in the world's population over the next decades [4], together with increased inclusion of seafood in diets [1], global demand for seafood is expected to increase [5]. Although, there may be some room for expansion of capture fisheries if fish stocks are rebuilt [6], production from capture fisheries has reached its maximum capacity in most parts of the global oceans (Fig 1) [7, 8]. Thus, increasing food production from aquaculture has been considered a main solution to meet the rising demand for seafood [9–11].

Aquaculture has grown rapidly over the last three decades, expanding at an average annual growth rate of 5.8% (2005–2014) [12], down from an average of 8.8% between 1980 and 2010 [13]. It is the fastest growing agro-food sector in the world [14]. In 2014, aquaculture contributed about 73.4 million tonnes (44%) to total food fish production, with about 27.6 million tonnes (37.6%) of this production from aquaculture in marine and brackish environment, also known as mariculture [12] (Fig 1). It is important to emphasise that more than 60% of this production stems from the farming of molluscs and crustaceans. Mariculture has expanded by 9.3% in production since 1990 [15, 16]. Currently, 112 countries and territories produce seafood in the marine environment [15] with earnings reaching 65.4 billion USD in 2013 from the mariculture sector [16] and representing 43.5% of the total aquaculture income. Given mariculture’s significant contribution to seafood supply and the economy, various attempts are being made to estimate the potential to expand aquaculture in the ocean [17]. Information on area potentially suitable for mariculture could be useful for planning ocean based activities (e.g. energy production, shipping, marine protected areas) [18, 19].

A range of environmental and social-economic factors influence the sustainable development of mariculture. Most mariculture involves growing fish or invertebrates in nets or cages that are submerged, allowing for free water exchange with the surrounding marine environment [20, 21]. Farmed species’ survival and growth rates are directly influenced by natural environmental conditions [22] that consequently affect the suitability of an area to farm these species. However, a number of other factors play an important role in determining the actual production capacity and its sustainability. These include but are not limited to: the environmental carrying capacity of the farming area [23], feed ingredients, e.g. the demand for forage fish in aquafeeds [8, 24, 25] or plant proteins [26], the development of closed life cycles farming techniques to reduce the need for wild-caught juveniles and broodstock for restocking [27, 28], other technological developments that reduce the environmental impacts of mariculture [29]; and the quality of governance to ensure sustainable mariculture practices [30].

An important first step towards better describing the ocean environmental spatial extent for sustainable mariculture production potential, is the identification of marine areas that offer suitable environmental conditions for currently farmed species. Since most ocean-based mariculture operations use open facilities in which farmed organisms are exposed to natural environmental conditions, the physical and chemical properties of the waters may thus affect the organisms’ growth and survivorship, and consequently, their potential for mariculture production. Here, suitable environmental conditions refer to the area of the ocean that can support the physiological needs of farmed species for sustainable mariculture production [31]. Environmental preferences of (farmed) marine species can be approximated and mapped using species distribution models (SDMs). Based on the environmental niche theory [32], this modelling approach consists of quantitatively describing the relationship between a species' observed occurrence records and various parameters that describe its environment. Such relationship can be developed using historical occurrence records of the species in both the natural and farmed environments. SDMs can be applied to predict species’ distribution in the past, present and future [33, 34].

The use of SDMs is particularly suited to marine ectotherms and thus have a spatial distribution that is tightly correlated to environmental conditions [35–37].

This study aims to predict the spatial extent of area that is suitable environmentally for mariculture. We applied four SDMs: Ecological Niche Factor Analysis (ENFA) [38], the Non- Parametric Probabilistic Ecological Niche (NPPEN) [39], Maximum Entropy (MAXENT) [40] and Surface Range Envelope (SRE) [41], to quantify the environmental niche of the presently important farmed species and project their habitat suitability index (HSI) over the global ocean based on current environmental conditions. We focused on coastal and open water farming systems only. Based on the predicted HSI, we calculated the total area of the world’s exclusive economic zones (EEZ) that is suitable for farming marine species. We examined the variation among models and compare it with the mean predictions across models to highlight where predictions were most robust to variations. We then evaluated model projection uncertainties and estimated the total area that would be suitable for mariculture. Finally, we discussed the implications of our results for future mariculture development.

## Material and methods

### 1. Biotic data

We obtained a list of farmed species from the Sea Around Us mariculture database (SAU, www.seaaroundus.org). The database is derived largely from the Food and Agricultural Organisation (FAO) database, with augmented information from national statistics to subdivide annual mariculture production by sub-national units (e.g., provinces, states), in addition to countries and taxa, for the period 1950 to 2010. We extracted the species’ names of all fish and invertebrates reported in the database (307 in total). Records that are not reported at the species level (i.e., with genus and species specified) were excluded from our analysis. Following the minimum occurrence data requirements for SDM [42], we only retained species that occurred in more than seven sub-national units. This is to ensure a greater model accuracy with higher numbers of occurrence locations. Thus, this study focused on a total of 102 species (57 Chordata, 15 Arthropods, 29 Mollusca and 1 Echinodermata) (S1 Table). Although seaweed mariculture contributes substantially to global production, we did not include seaweed in this study because of the lack of geospatial data on seaweed mariculture locations. Species’ ecological information, including maximum depth, minimum depth, trophic level, preferred biome [43] and habitat, were obtained from FishBase (http://www.fishbase.org/) and the Encyclopaedia of life (http://eol.org/).

To obtain representative spatial distribution of each farmed species and quantify their environmental niche, we developed two databases. The first one consisted of natural occurrence records (i.e., from the wild) for all 102 species from a number of open source databases: Ocean Biogeographic Information System (OBIS, http://www.iobis.org/), Global Biodiversity Information Facility (GBIF, http://www.gbif.org/), FishBase (http://www.fishbase.org/) and the International Union for the Conservation of Nature (IUCN, http://www.iucnredlist.org/technical-documents/spatial-data). For each species, we removed duplicate records of occurrences among databases and records for which geographic information was not available.

Secondly, we developed a georeferenced occurrence database exclusively for mariculture (i.e., coastal and open ocean farmed). Using all sub-national units where farms were recorded in the Sea Around Us mariculture database, we visually identified any mariculture installations (pens, cages and lines) based on satellite photos available from Google Earth (http://www.google.com/earth/). The coordinates were extracted for each installation by using the Google Earth placemark tool [44].

All species’ natural occurrence and mariculture location records were converted to a binary database of presence or absence and rasterized on a regular spatial grid of 0.5° latitude by 0.5° longitude over the global ocean.

### 2. Environmental data

We collected eight environmental parameters: temperature, dissolved oxygen concentration, chlorophyll-*a* concentration, salinity, pH, silicate concentration, current velocity and euphotic depth. Annual climatology for the period 1955–2012 for temperature, salinity, dissolved oxygen concentration and silicate concentration were obtained from the World Ocean Atlas 2013 (http://www.nodc.noaa.gov/OC5/woa13/). Euphotic depth and chlorophyll-*a* concentration annual climatology for the period from 1998 to 2012 were downloaded from the Ocean Colour website (http://oceancolor.gsfc.nasa.gov). We obtained 10-year averaged ocean current velocity data (1992–2002) from the Estimating the Circulation and Climate of the Ocean (ECCO) Project (http://www.ecco-group.org). Surface and bottom pH values were extracted from the Geophysical Fluid Dynamics Laboratory Earth System Model (GFDL-ESM-2G) and averaged over the period 1970–2000. All environmental data were interpolated using bilinear methods [45] over the global ocean (189.75°W to 179.75°E and 89.75°N to 89.75°S) on a regular spatial grid of 0.5° latitude by 0.5^o^ longitude (the same as occurrence rasterized data) and for two vertical layers: surface (0-10m) and sea bottom depth where available.

### 3. Modelling habitat suitability

We predicted the habitat suitability for each farmed species on 0.5^o^ by 0.5^o^ grid of the global ocean using species distribution models (SDMs). Firstly, we harmonised the biotic and environmental data based on the regular spatial grid coordinates with data on occurrences in natural and mariculture environments. Secondly, we determined the most important environmental parameters to model the farmed marine species’ distribution using the eigenvalue diagram implemented in Ecological Niche Factor Analysis (ENFA) [38]. The diagram was constructed based on the departure of the ecological niche from the mean habitat for each species, thus identifying the species’ preference for particular environmental parameters among the whole set of parameters (S1 Fig). We selected the most important set of environmental parameters by identifying the direction in which the ‘specialisation’ was highest. Specialisation is a measure of the narrowness of the niche, i.e., the higher the specialisation, the more restricted the niche. We then used these selected parameters for all SDM in this study. Finally, we predicted the habitat suitability for each species (mariculture and natural occurrence) using four SDMs. A multiple-model approach was used to explore the variations and uncertainty of predictions from different SDMs [37, 46].

The four SDMs predict species distributions based on different algorithms and assumptions. The first SDM, ENFA, is an analysis that uses multivariate statistics to assess species’ habitat selection by providing the realised niche measurement within the available hyper-space from two estimates: the marginality, that identifies a species’ preference for given environmental conditions, and its specialisation (i.e., the species’ sensitivity to variations in its optimum environment) [38].The lower the marginality, the less the niche deviates from available conditions; the higher the specialisation the more restricted the niche [47]. The second model, the Non- Parametric Probabilistic Ecological Niche (NPPEN) [39], is based on a simplification of the Multiple Response Permutation Procedures (MRPP) using the Generalised Mahalanobis distance. The other two SDMs, Maximum Entropy (MAXENT) [40] and Surface Range Envelope (SRE) [41], use various numerical procedures. MAXENT estimates the ratio of a species’ presence site to the study area and then calculates the probability of occurrence through a logistic transformation [48]. SRE is an environmental envelope model that identifies cells, which have environmental values that fall within the range of values measured from the presence data [49]. All the SDMs use presence-only data to determine a species’ environmental distribution. We applied each SDM to predict a species’ habitat suitability index (HSI)—an index that scales from 0 to 1 to indicate the environmental suitability of the selected environmental conditions in each spatial cell for each studied species.

### 4. Model testing

We tested the robustness of the SDM outputs by comparing the predicted HSI with reference records of species occurrences. Mariculture and natural occurrence data were analysed separately and divided into two datasets: 75% of all records were used for training purposes to develop each SDM and calculate each species’ HSI. The remaining 25% of the data were used for model evaluation. Specifically, the Area Under the Receiver Operating Characteristic Curve (AUC) of each set of model predictions was calculated using the ROCR package in R [50]. The AUC values range from 0 to 1, with 0.5 indicating that the model is no better than a random sample of values and 1 indicating that the model has high predictive power.

We examined the correlation between the HSI predictions from the natural and mariculture occurrence data. For each species, we fit a linear model to predicted HSI from mariculture and natural occurrences as dependent and independent variables, respectively. We cross-validated this statistical relationship by applying a generalised linear model to the combined species HSI by assuming binomial error distribution. We calculated the degree of overdispersion for proportional data from residue deviance and then fitted a quasibinomial distribution to account for the overdispersion [51]. We set a minimum predicted HSI for each species as the value below which a species was considered to be absent. This approach used species-specific minimum HSI thresholds instead of a fixed threshold for all species, as used by Jones and Cheung [37]. The thresholds were identified by quantifying a species’ “prevalence” (i.e., the fraction of cells at which the species is present) [52] and represent the minimum HSI threshold for that species. This tests mariculture location HSI (presence) versus natural occurrence HSI (absence). The predictive values of habitat suitability for mariculture below the prevalence value were assigned 0, and predicted HSI values higher than the prevalence value were assigned 1.

### 5. Identifying potential mariculture area

The first criterion that we used to define potential mariculture area was the suitability of the environment for farmed species. For an area to be suitable for mariculture, it needs to meet the minimum environmental conditions for the growth and survival of farmed species. Thus, for each farmed species, its potential mariculture area must have an HSI above its minimum threshold (i.e., prevalence).

Secondly, we assumed that mariculture operations would not go beyond the area of sovereign nations’ jurisdiction, and that water current and ocean management would be gruelling operations. Thus, we constrained the potential mariculture area to be within countries’ Exclusive Economic Zone (EEZ). We also assumed that mariculture could only be found in spatial cells with a current velocity between 10 cm.s^-1^ and 100 cm.s^-1^ [53]. Low current would result in more rapid food depletion (less particulate organic matter flow) and less efficient production [54], less waste removal (feed and organic waste), and high benthic impact in finfish aquaculture [55]. In contrast, strong currents can damage farm structures and holding facilities [21] and affect the growth of farmed fish through skeletal malformations [56]. Furthermore, as most marine protected areas (MPAs) do not allow mariculture activities to take place, they were not considered as suitable future mariculture areas (Table 1).

**Table 1:** Selected criteria for defining the potential area for mariculture production.

Justification	Criteria	Threshold
Political boundary	Within exclusive economic zone (EEZ)	200nm (370.4 km)
Disturbance by strong ocean currents	Within a range of ocean suitable current velocities	Current velocity between 10 cm.s^-1^ and 100 cm.s^-1^
Conflicting use of waters	Area outside of marine protected area	Potential area exclude marine protected sites

We calculated the total suitable mariculture area for the 102-farmed species considered for each model and derived an overall multi-model weighted average (each model contributed to the final output based on its AUC value). We also calculated the number of species that were predicted to be suitable for mariculture in each spatial cell. We then compared the spatial distribution of potential mariculture area and species richness of the 20-farmed species with the highest cumulative production from 1950 to 2010.

## Results

### 1. Prediction of HSI

**Fig 2:**
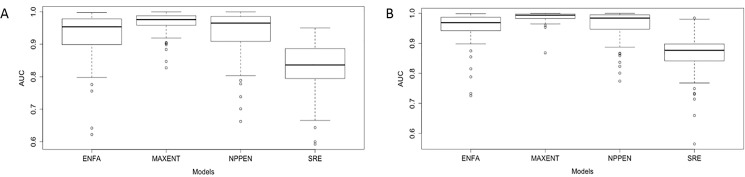
Prediction evaluation of each SDM used in the analysis. (A) AUC for the habitat suitability index (HSI) for natural occurrences of farmed species across SDM; (B) AUC for mariculture location HSI across SDMs. The horizontal lines represent median values. The upper and lower boundaries of the box represent the upper and lower quartiles of the data. ENFA- Ecological Niche Factor Analysis, MAXENT- Maximum Entropy, NPPEN- Non- Parametric Probabilistic Ecological Niche and SRE- Surface Range Envelope.

The predicted mariculture and natural habitat suitability index generally agree with observed occurrences. Almost all predicted species distributions for all species and models had AUC values greater than 0.7 (Fig 2). The AUC values varied among models, with predictions from SRE having a median AUC across species < 0.8, and predictions from MAXENT scoring the highest median AUC values. The 25^th^ and 75^th^ percentiles of the estimated prevalence value for each species ranged from 0.30 to 0.62.

### 2. Potential mariculture area and species richness

**Fig 3:**
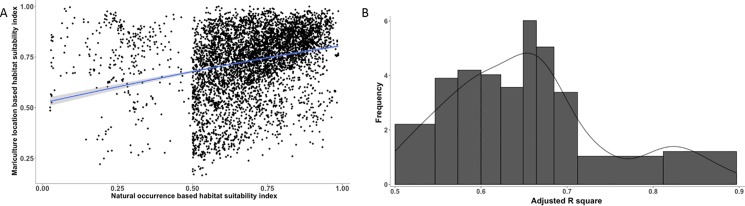
The relationship between predicted mariculture habitat suitability index (HSI) and natural occurrence habitat suitability index. (A) Regression of global predicted mariculture HSI and natural occurrence HSI (p<0.001). (B) Histogram of adjusted R^2^ of individual species’ regression analysis with a mean value of 0.66.

Overall, we found a significant and positive relationship between the environmental niche of mariculture locations and that of farmed species’ natural occurrences. Across all farmed species, we found a significant and positive linear relationship between mariculture occurrence-based (y) and natural occurrence-based (x) HSI (y = 0.27x + 0.54, p < 0.001) (Fig 3A). For each individual species, the adjusted R-square values of the regression between mariculture and natural-based HSI were above 0.50, with a mean of 0.66 (Fig 3A). Species that showed the strongest relationship between the mariculture-based and natural-based HSI were Red drum (*Sciaenops ocellatus*), Peruvian scallop (*Argopecten purpuratus*) and Pacific cupped oyster (*Crassostrea gigas*) (R^2^ = 0.90, 0.88 and 0.86, respectively).

**Fig 4:**
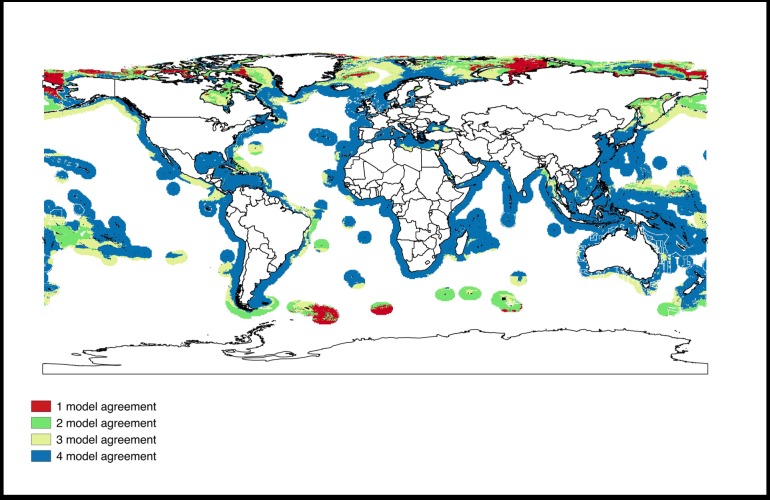
Predicted suitable marine area for mariculture and the agreement among SDMs. Blue—high agreement (4 models), Yellow—moderate agreement (3 models), Green—low agreement (2 models) and Red–very low agreement (1 model).

**Fig 5:**
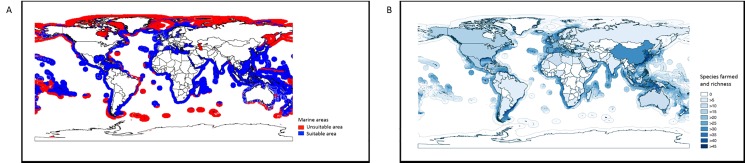
Potential marine area suitable for mariculture production and current versus potential farmed species richness. (A) Total predicted suitable marine areas for mariculture in blue and unsuitable marine areas in red based on an average of four different species distribution models; (B) Comparison between present numbers of species farmed in different countries with potential numbers of farmed species based on model outputs.

**Fig 6:**
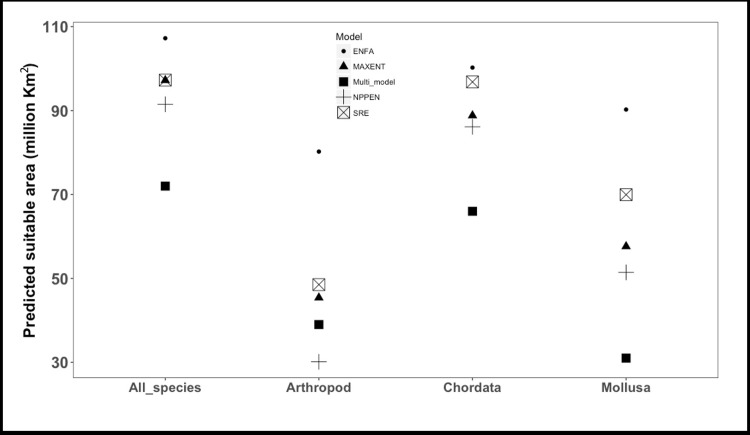
Predicted marine area suitable for mariculture from four species distribution models: ENFA, MAXENT, NPPEN and SRE; and weighted Multi-model. ENFA predicted the highest area with 107 million km^2^ while the Multi-model predicted 72 million km^2^.

The predicted environmentally suitable areas for mariculture with high agreement among the four SDMs are found between 66.5^o^ N and 66.5^o^ S (Fig 4). Based on the weighted average predictions from the four SDMs, the total suitable mariculture area for the 102 species was estimated at 72 million km^2^ (Fig 5A). Predictions from ENFA result in the largest suitable marine area (107.0 million km^2^) while NPPEN yielded the lowest value (91.5 million km^2^) (Fig 6). Sixty-six million km^2^ of this area is suitable for finfish, 39 million km^2^ for crustaceans and 31 million km^2^ for mollusc. This included areas currently used for mariculture purposes. Areas that were predicted as unsuitable for the mariculture of any species include Antarctica and pockets around the Arctic region.

Mariculture species richness was highest in potential mariculture areas between 25° N and 25° S (S4 Fig) and include the southwestern Atlantic coast (species richness = 30–45 among models) and West Africa (species richness = 35–40 among models). Other notable areas with high mariculture species richness included the Gulf of Mexico, the Caribbean Sea, the East China Sea, the Yellow Sea, the Sea of Japan and the Banda Sea off the coast of Timor-Leste. However, despite results showing high species richness for these potential mariculture areas, most of the species were not being farmed in these regions.

**Fig 7:**
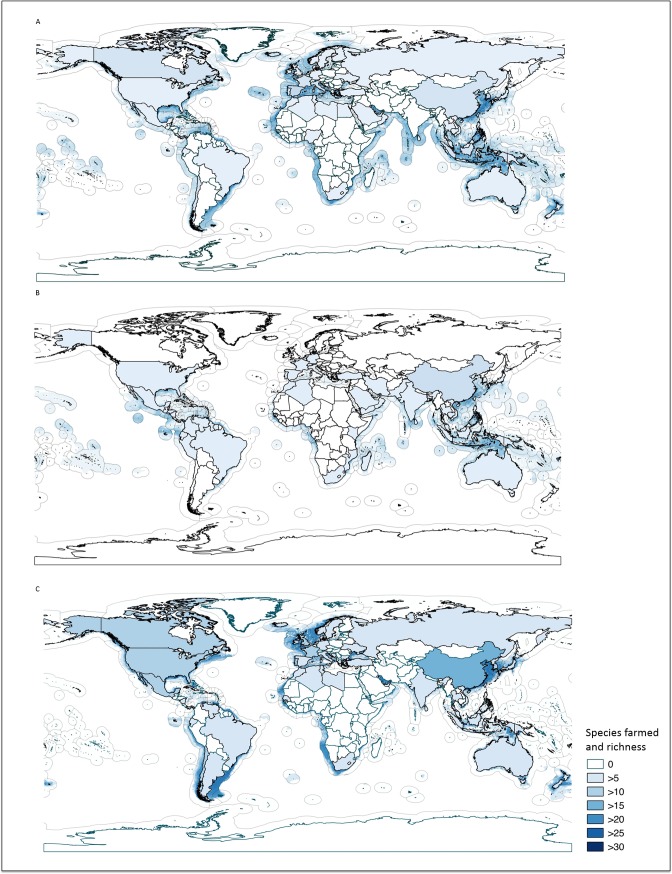
**Global predicted potential mariculture area and regional farmed species richness for** (A) finfish (66 million km^2^); (A) crustaceans (39 million km^2^); and (C) molluscs (31 million km^2^).

Specifically, we found a large potential area for finfish mariculture in the tropics, between 20°N and 20°S (mariculture species richness between 5 and 30 spp.) (Fig 7). In contrast, molluscs were predicted to be the dominant mariculture group in the temperate areas (23.0° to 66.5° in both the northern and southern hemispheres), with mariculture species richness of 3 to 15 spp. Large potential areas for crustacean mariculture were predicted in waters within 23° N to 25° N and 23° S to 25° S, with predicted mariculture species richness of 2 to 12 spp. and 3 to 11 spp., respectively.

### 3. Species and country-level comparisons

**Fig 8:**
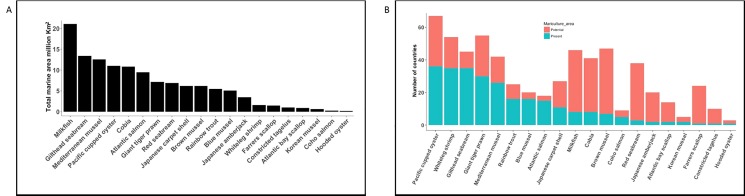
Total predicted potential mariculture area for 20 of the most cultured species based on cumulative production from 1950–2010. (A) Classification by total potential marine areas suitable for farming. (B) Classification by total present and potential number of countries.

Predicted potential mariculture area varied across the most important farmed species. Milkfish (*Chanos chanos*) was predicted to have the largest suitable farming area (21 million km^2^), while hooded oyster (*Saccostrea cucullata*) was predicted to have the smallest area (0.2 million km^2^) amongst the 20-species selected for comparison (Fig 8A). We found a minimum of 10% difference between the number of countries currently practising mariculture and those with the potential for marine farming production. For example, predicted mariculture potential area was 16% larger than the area where mariculture was reported to have occurred for Atlantic salmon (*Salmo salar*). For Cobia (*Rachycentron canadum*) and Pacific cupped oyster (*Crassostrea gigas*), reported mariculture area was 80% and 46% below their predicted spatial extent, respectively.

## Discussion

### 1. Overall model performance

By using an ensemble of SDMs [37], we were able to explore the structural uncertainty of the predicted mariculture species distributions and environmental suitability. Projections from ENFA and SRE showed considerable variation in comparison with MAXENT and NPPEN. MAXENT and NPPEN had the highest AUC values. For the former approach, this may be due to the model’s weighting ability of input variables [37], thereby preventing over-fitting of the model by determining how much the likelihood should be penalised [57]. NPPEN measures the correlation between species’ occurrence and distribution, thus avoiding random quantile classes selection to allow direct estimation of species occurrence [39]. The multi-SDM approach explored the uncertainty due to discrepancies among models thereby capturing the full range of potential suitable marine areas for mariculture.

The relevance of the set of environmental factors identified by the modelling algorithms applied here to best predict mariculture areas are supported by empirical knowledge [58–61]. For example, the physiology of farmed species is affected by changes in temperature [62]. Optimal temperature determination for farmed species growth is important practice in mariculture [63]. In addition, oxygen is an important factor affecting the growth of fishes [64], and dissolved oxygen level is an important consideration in determining the holding capacity of fish farms [65, 66]. Salinity influences the growth some of farmed species, with salinity levels above 25 Practical Salinity Unit (PSU) having shown sometimes to result in high mortality and retarded growth in cultured scallop (*pecten maximus*) [67, 68] while such salinity level could yield better growth performance in seabream [69]. Moreover, primary production and suspended particulate organic matter or seston, indicated by chlorophyll *a* concentrations and silicate, respectively, are food sources for molluscs [70, 71]. Thus, their concentration is expected to affect the growth of farmed molluscs.

### 2. Mariculture opportunities and limitations

Overall, our findings suggest that the global ocean environmentally suitable area for mariculture is much larger than the area in which mariculture is currently practised. Particularly, most offshore areas considered environmentally suitable are not being used for farming activities. Our results therefore suggest that the lack of environmentally suitable area for mariculture is not the main limiting factor for the expansion of mariculture [30] in most regions of the world. Instead, other factors such as the socio-economics of producing countries, including capacity and political instability; technology, its availability and cost effectiveness [72]; trades; aqua feed availability [73, 74]; aquaculture development-related policies [75, 76] and competition for space within an EEZ for instance; shipping, oil and gas, as well as tourism all play major roles in the development of mariculture operations and their future expansion.

Our conclusion about the area suitable for mariculture and its limitation for its utilization for mariculture operations agree with a related study that employed different methods to estimate the global potential area for mariculture [17]. Specifically, Gentry *et al*. [17] applied more constraints than our study in estimating available ocean area for mariculture, resulting in a lower estimate. For instance, our study does not consider water depth as a limiting factor to define environmentally suitable areas for finfish, as the use of submersible cages is widespread for a number of farmed species (e.g., Salmon, Cobia) [77]. In this case feed development, socio-economic and technological factors may represent a constraint to farming rather than water depth itself [78]. The use of multiple approaches to predict suitable mariculture area should be encouraged so that the uncertainties associated with such predictions can be better characterised.

Regionally, our results show that the difference between the predicted environmentally suitable area for mariculture and the extent of current mariculture activities is largest in Africa, the Caribbean and along the Atlantic coast of South America. These regions are predicted hotspots for mariculture species richness (60% of the 102 species), yet actual mariculture operations appear to be relatively limited, accounting for only 1.3% of global mariculture production [15, 16]. Factors such as poor economic conditions, lack of supporting infrastructure [79], political instability, limited foreign investment in the sector [80] and inadequate value chain linkages [26] in many countries of these regions may have impeded mariculture development.

China, in contrast, is presently using the largest extent of its suitable area for marine aquaculture. The country has a long history of aquaculture, going back 2500 years [81]. Owing to the late 70s economic reform initiatives, China’s aquaculture industry has benefited from open market policies. In addition, the fact that China accounts for one- quarter of the global fish demand has made the country’s market suitable for expansion. However, farm expansion might exert more pressure on wild capture fisheries as China’s aquaculture industry accounts for one- third of global fishmeal production [82].

Concerns regarding the broader environmental sustainability of mariculture may limit the sector’s expansion. Particularly, the expansion of farming carnivorous species will increase the demand for fish resources in feeds, adding to the stress on forage fisheries and also other non-selective trawling [24]. Fishmeal and oil are reduction fisheries products and the two most essential inputs into aqua feed since they are highly digestible and essential sources of amino acids [83, 84]. While successful partial replacement of fishmeal with plant and other sources (e.g. insects, yeast and algae) has been tested and used in aqua feed [85, 86], there are still challenges remaining for using fully plant-based feed source particularly for high value species such as Atlantic salmon [74]. High quality plant based ingredients used in feeds must also in the long run be replaced by innovative resources not competing with human food.

Affordable and efficient technology can also contribute to the sustainable expansion of mariculture. Significant advances have been achieved in land-based systems designed to reduce nutrient load discharges and farmed species escapes, as well as improve disease management [87–89]. In coastal finfish and shellfish mariculture systems, a variety of cages are being developed to withstand high wave actions and reduces escapes in offshore areas [21, 90]; advances that will be necessary given predictions of increased storm activity in the future [91, 92]. For crustaceans, such advances are lagging behind and a number of significant hurdles will need to be overcome for these to become operational. In addition, integrated multi-trophic aquaculture systems (IMTA) where lower trophic level species, such as seaweed and bivalves, are farmed together with finfish [29] maximise inorganic and organic nutrient waste recycling, thereby reducing the environmental footprint of mariculture farms. While these systems hold much promise, large commercial IMTA farms are still uncommon.

Strong environmental governance is also needed to regulate and ensure the sustainable development of mariculture. Aquaculture activities are regulated by law in many countries (e.g., Canada, China, Norway and the Philippines [93]). Effective monitoring and enforcement are imperative if mariculture expansion is to be sustainable in the future. However, the monitoring and enforcement varies considerably among countries [94]. Countries such as the U.S. [95], Australia [96], Canada and the E.U. [97] have also adopted formal codes of conduct whose main objective is to promote the responsible development and management of aquaculture with appropriate sanctions.

The high correlation for individual species’ predicted HSI between mariculture location and natural occurrence in this study, suggests that mariculture farms are sensitive to changes in environmental conditions driven by climate change or other anthropogenic activities such as pollution [35, 36, 98–100]. Ocean warming may drive environmental conditions beyond the suitable range for mariculture and will cause thermal stress for a number of currently farmed species (e.g., cod, oysters). However, these rising temperatures may extend the the growing season for some species and may provide opportunities to farm new species, or species that are currently economically marginal in the affected areas. Also, shellfish aquaculture is sensitive to ocean acidification [101–103], as lower carbonate saturation state in the water can make it more difficult for calcifying invertebrates to produce shells. Carbon emissions may thus have a substantial impact on the distribution and diversity of potentially suitable farm sites for currently farmed species.

## Conclusion

In this study, we identified a large global environmentally suitable area for mariculture. We suggest that other non-environmental factors such as technological, economic and social constraints play a fundamental role in determining mariculture production in these regions. While, our approach is useful in defining areas on a broad scale, more detailed studies on the ‘ecological suitability’ (i.e., in terms of carrying capacity) will be required to further constraint the prediction of suitable area for mariculture development. Also, these currently suitable areas might transition to being unsuitable in the future due to human activities such as pollution, coastal zone activities, and climate change. Future studies should include other human uses of marine areas e.g. ship routes, wind farms etc. and their potential competition with mariculture to further characterize the suitable areas for farming. Moreover, given the importance of seaweed farming in various regions, future studies should also collate data and information about seaweed mariculture locations and extend all analyses to seaweed. It will also be important to investigate and address the main constraints in sustainable mariculture development to help develop pathways that ensure the continued contribution of mariculture to global seafood production.

## Supporting information

S1 Table List of species in this study. (DOC)

S1 Fig ENFA biplot with the x-axis (marginality) and y-axis (specialisation). **The white dot within the dark area represents the centre of used area while the light area is the available niche. The arrows are projections of oceanic parameters based on mariculture locations of the species** (A) Pacific cupped oyster (*Crassostrea gigas*) (B) Cobia (*Rachycentron canadum*) (C) Atlantic salmon (*Salmo salar*) (D) Giant tiger shrimp (*Penaeus monodon*).
(TIF)

S2 Fig Positive linear relationship between mariculture occurrence based (y) and natural occurrence based (x) HSI (y = 0.27x + 0.54, p < 0.001). (TIF)

S3 Fig The linear regression between predicted mariculture location and natural occurrence habitat suitability. (A) Pacific cupped oyster (*Crassostrea gigas*) y = 0.01773x + 0.7906, R^2^ = 0.8607, p < 0.0001.
(B) Cobia (*Rachycentron canadum*) y = 0.04762x + 0.5525, R^2^ = 0.5951, p < 0.0001.
(C) Atlantic salmon (*Salmo salar*) y = 0.03001x + 0.7027, R^2^ = 0.8118, p < 0.0001.
(D) Giant tiger shrimp (*Penaeus monodon*) y = 0.02476x + 0.7012, R^2^ = 0.7383, p < 0.0001.
(TIF)

S4 Fig Latitudinal predicted species richness. (A) global mariculture species richness (B) Finfish (C) Crustacean (D) Molluscs
(TIF)

S5 Fig Suitable marine areas for farming. (A) Pacific cupped oyster (*Crassostrea gigas*) (B) Cobia (*Rachycentron canadum*) (C) Atlantic salmon (*Salmo salar*) (D) Giant tiger shrimp (*Penaeus monodon*).
(TIF)
