# Supplementary material for: Global estimation of areas with suitable environmental conditions for mariculture species
Source: PLoS One. 2018 Jan 19;13(1):e0191086. doi: 10.1371/journal.pone.0191086 (PMC5774971; doi:10.1371/journal.pone.0191086)
Supplement: S1 Table — (DOC) [file pone.0191086.s001.doc]

**Global estimation of areas with suitable environmental conditions for mariculture species.**

**Supporting Information**

Authors: Muhammed A. Oyinlola^1*^, Gabriel Reygondeau^1^, Colette C.C. Wabnitz^1^,

Max Troell ^2,3^ and William W.L. Cheung^1^

^1^ Nippon Foundation-Nereus Program and Changing Ocean Research Unit, Institute for the Oceans and Fisheries, The University of British Columbia, Vancouver, Canada

^2^ Stockholm Resilience Centre, Stockholm University, Stockholm, Sweden

^3^ The Beijer Institute, The Swedish Royal Academy of Sciences, Stockholm, Sweden

**S1 Table: List of species in this study**

| ID | Species | Phylum | Environment |
| --- | --- | --- | --- |
| 1 | *Acanthopagrus schlegeli* | Chordata | Marine/ Brackish |
| 2 | *Anguilla anguilla* | Chordata | Marine/ Brackish |
| 3 | *Anguilla bicolor bicolor* | Chordata | Marine/ Brackish |
| 4 | *Apostichopus japonicus* | Echinodermata | Marine |
| 5 | *Argopecten irradians* | Mollusca | Marine |
| 6 | *Argopecten purpuratus* | Mollusca | Marine/ Brackish |
| 7 | *Argyrosomus regius* | Chordata | Marine/ Brackish |
| 8 | *Cerastoderma edule* | Mollusca | Marine |
| 9 | *Chanos chanos* | Chordata | Marine/ Brackish |
| 10 | *Chlamys farreri* | Mollusca | Marine |
| 11 | *Coregonus lavaretus* | Chordata | Marine/ Brackish |
| 12 | *Crassostrea gigas* | Mollusca | Brackish |
| 13 | *Crassostrea rhizophorae* | Mollusca | Brackish |
| 14 | *Crassostrea virginica* | Mollusca | Brackish |
| 15 | *Decapterus macrosoma* | Chordata | Marine |
| 16 | *Dentex dentex* | Chordata | Marine |
| 17 | *Dicentrarchus labrax* | Chordata | Marine/ Brackish |
| 18 | *Diplodus puntazzo* | Chordata | Marine/ Brackish |
| 19 | *Epinephelus coioides* | Chordata | Marine/ Brackish |
| 20 | *Epinephelus fuscoguttatus* | Chordata | Marine/ Brackish |
| 21 | *Epinephelus malabaricus* | Chordata | Marine/ Brackish |
| 22 | *Epinephelus polyphekadion* | Chordata | Marine |
| 23 | *Epinephelus septemfasciatus* | Chordata | Marine |
| 24 | *Epinephelus tauvina* | Chordata | Marine |
| 25 | *Gadus morhua* | Chordata | Marine/ Brackish |
| 26 | *Haliotis discus* | Mollusca | Marine |
| 27 | *Haliotis diversicolor* | Mollusca | Marine |
| 28 | *Haliotis rufescens* | Mollusca | Marine |
| 29 | *Halocynthia roretzi* | Chordata | Marine |
| 30 | *Helicolenus dactylopterus dactylopterus* | Chordata | Marine |
| 31 | *Hippoglossus hippoglossus* | Chordata | Marine |
| 32 | *Lateolabrax japonicus* | Chordata | Marine/ Brackish |
| 33 | *Lates calcarifer* | Chordata | Marine/ Bbrackish |
| 34 | *Lutjanus argentimaculatus* | Chordata | Marine/ Brackish |
| 35 | *Lutjanus ehrenbergii* | Chordata | Marine/ Brackish |
| 36 | *Lutjanus johnii* | Chordata | Marine/ Brackish |
| 37 | *Mercenaria mercenaria* | Mollusca | Marine |
| 38 | *Meretrix meretrix* | Mollusca | Marine |
| 39 | *Metapenaeus ensis* | Arthropod | Marine/ Brackish |
| 40 | *Mugil cephalus* | Chordata | Marine/ Brackish |
| 41 | *Acanthopagrus latus* | Chordata | Marine/ Brackish |
| 42 | *Mytilus chilensis* | Mollusca | Brackish |
| 43 | *Mytilus coruscus* | Mollusca | Marine |
| 44 | *Mytilus edulis* | Mollusca | Marine/ Brackish |
| 45 | *Mytilus galloprovincialis* | Mollusca | Marine |
| 46 | *Oncorhynchus kisutch* | Chordata | Marine/ Brackish |
| 47 | *Oncorhynchus mykiss* | Chordata | Marine/ Brackish |
| 48 | *Oncorhynchus tshawytscha* | Chordata | Marine/ Brackish |
| 49 | *Ostrea edulis* | Mollusca | Marine/ Brackish |
| 50 | *Pagellus bogaraveo* | Chordata | Marine |
| 51 | *Pagrus major* | Chordata | Marine |
| 52 | *Pagrus pagrus* | Chordata | Marine |
| 53 | *Panulirus ornatus* | Arthropod | Marine |
| 54 | *Paralichthys olivaceus* | Chordata | Marine |
| 55 | *Patinopecten yessoensis* | Mollusca | Marine |
| 56 | *Pecten maximus* | Mollusca | Marine |
| 57 | *Fenneropenaeus chinensis* | Arthropod | Brackish |
| 58 | *Penaeus indicus* | Arthropod | Marine/ Brackish |
| 59 | *Penaeus japonicus* | Arthropod | Marine/ Brackish |
| 60 | *Penaeus merguiensis* | Arthropod | Marine/ Brackish |
| 61 | *Penaeus monodon* | Arthropod | Marine/ Brackish |
| 62 | *Penaeus penicillatus* | Arthropod | Marine/ Brackish |
| 63 | *Penaeus semisulcatus* | Arthropod | Marine/ Brackish |
| 64 | *Litopenaeus stylirostris* | Arthropod | Marine/ Brackish |
| 65 | *Penaeus vannamei* | Arthropod | Marine/ Brackish |
| 66 | *Perna perna* | Mollusca | Marine/ Brackish |
| 67 | *Perna canaliculus* | Mollusca | Marine |
| 68 | *Perna viridis* | Mollusca | Brackish |
| 69 | *Pinctada fucata* | Mollusca | Marine |
| 70 | *Pinctada maxima* | Mollusca | Marine/ Brackish |
| 71 | *Plectropomus leopardus* | Chordata | Marine |
| 72 | *Polydactylus sexfilis* | Chordata | Marine/ Brackish |
| 73 | *Polydactylus plebeius* | Chordata | Marine/ Brackish |
| 74 | *Portunus pelagicus* | Arthropod | Marine/ Brackish |
| 75 | *Portunus trituberculatus* | Arthropod | Marine |
| 76 | *Rachycentron canadum* | Chordata | Marine/ Brackish |
| 77 | *Ruditapes decussatus* | Mollusca | Marine |
| 78 | *Ruditapes philippinarum* | Mollusca | Brackish |
| 79 | *Salmo salar* | Chordata | Marine/ Brackish |
| 80 | *Salmo trutta fario* | Chordata | Marine/ Brackish |
| 81 | *Salvelinus alpinus* | Chordata | Marine/ Brackish |
| 82 | *Salvelinus fontinalis* | Chordata | Marine/ Brackish |
| 83 | *Sciaenops ocellatus* | Chordata | Marine/ Brackish |
| 84 | *Scomber japonicus* | Chordata | Marine |
| 85 | *Scophthalmus maximus* | Chordata | Marine/ Brackish |
| 86 | *Scylla serrata* | Arthropod | Marine/ Brackish |
| 87 | *Seriola dumerili* | Chordata | Marine |
| 88 | *Seriola quinqueradiata* | Chordata | Marine |
| 89 | *Siganus guttatus* | Chordata | Marine/ Brackish |
| 90 | *Sinonovacula constricta* | Mollusca | Marine |
| 91 | *Solea senegalensis* | Chordata | Marine |
| 92 | *Solea solea* | Chordata | Marine/Brackish |
| 93 | *Sparus auratus* | Chordata | Marine/ Brackish |
| 94 | *Takifugu chinensis* | Chordata | Marine |
| 95 | *Thunnus maccoyii* | Chordata | Marine |
| 96 | *Thunnus thynnus* | Chordata | Marine/ Brackish |
| 97 | *Trachurus japonicus* | Chordata | Marine |
| 98 | *Tridacna squamosa* | Mollusca | Marine |
| 99 | *Venerupis pullastra* | Mollusca | Marine |
| 100 | *Metapenaeus dobsoni* | Arthropod | Marine |
| 101 | *Saccostrea cuccullata* | Mollusca | Marine |
| 102 | *Sebastes schlegeli* | Chordata | Marine |


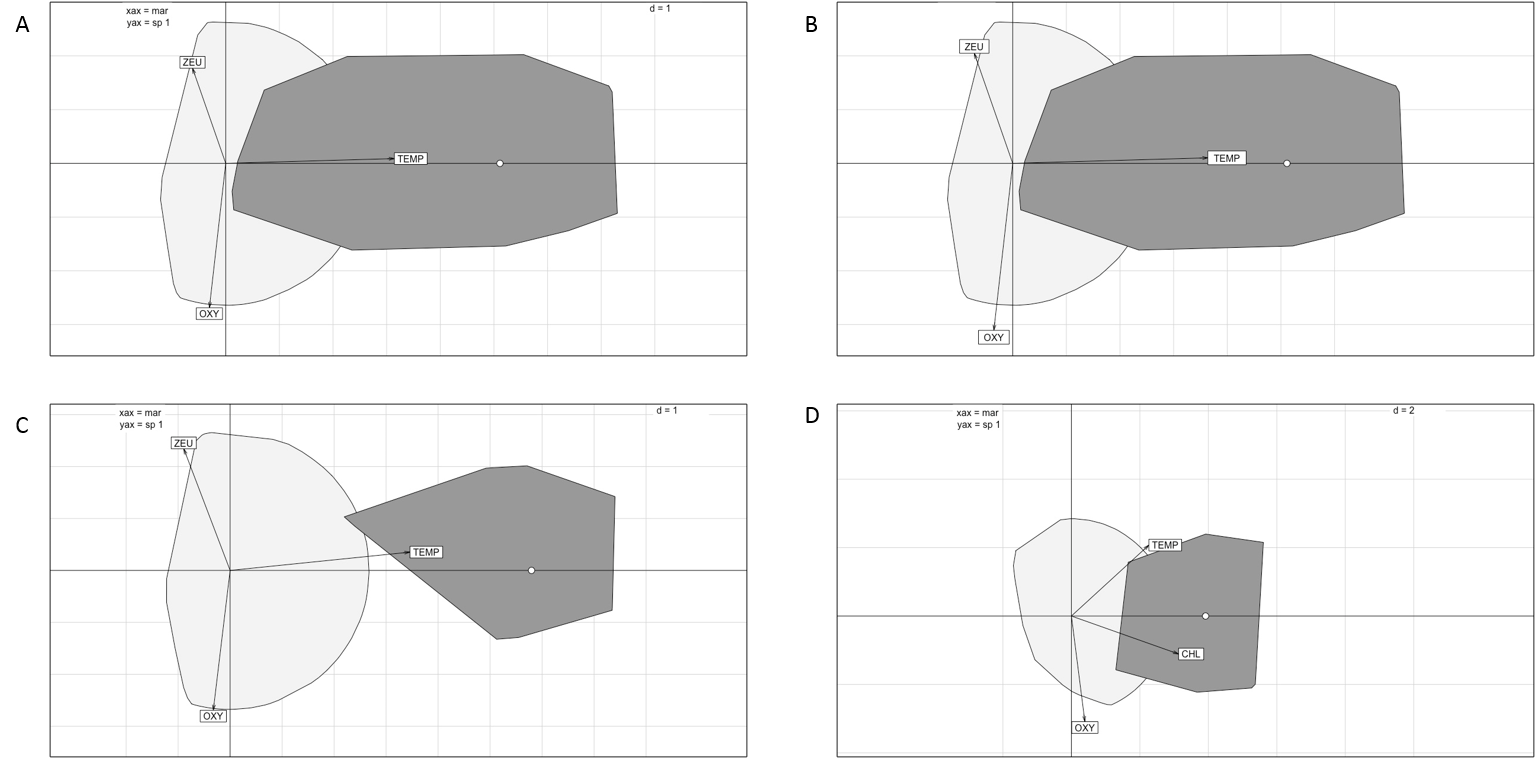


**S1 Fig: ENFA biplot with the x-axis (marginality) and y-axis (specialisation). The white dot within the dark area represents the centre of used area while the light area is the available niche. The arrows are projections of oceanic parameters based on mariculture locations of the species** (A) Pacific cupped oyster (*Crassostrea gigas*) (B) Cobia (*Rachycentron canadum*) (C) Atlantic salmon (*Salmo salar*) (D) Giant tiger shrimp (*Penaeus monodon*)


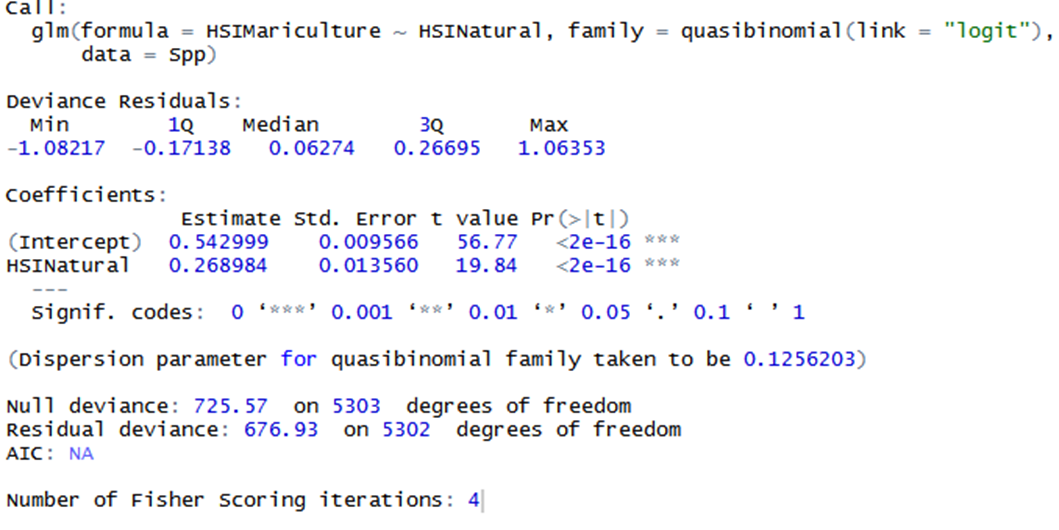


**S2 Fig**: Positive linear relationship between mariculture occurrence based (y) and natural occurrence based (x) HSI (y=0.27x + 0.54, p < 0.001)


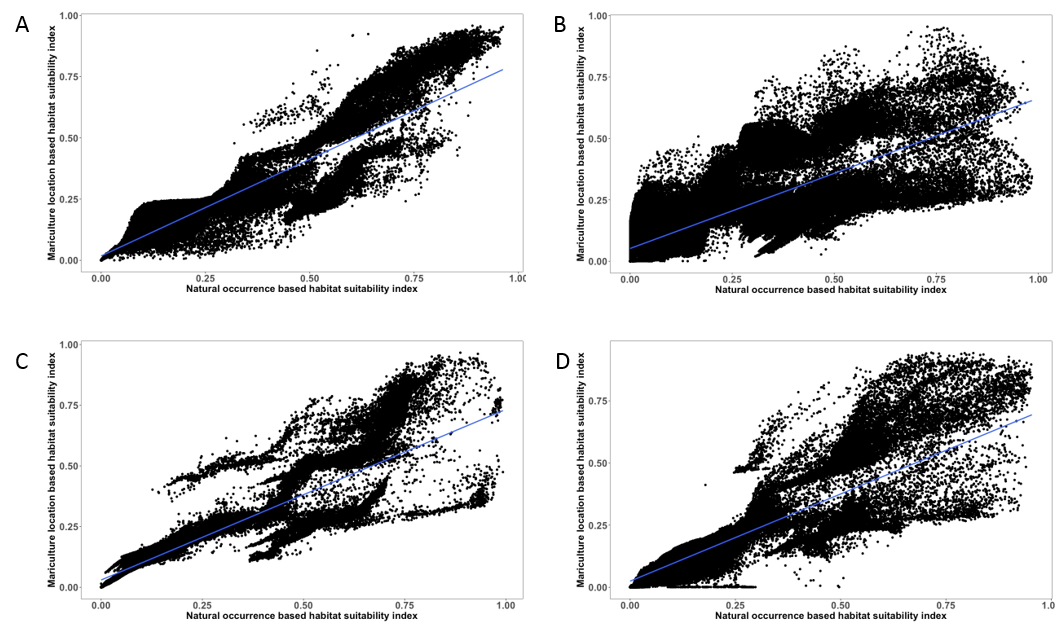


**S3 Fig: The linear regression between predicted mariculture location and natural occurrence habitat suitability** (A) Pacific cupped oyster (*Crassostrea gigas*) y=0.01773x + 0.7906, R^2^ = 0.8607, p < 0.0001

(B) Cobia (*Rachycentron canadum*) y=0.04762x + 0.5525, R^2^ = 0.5951, p < 0.0001

(C) Atlantic salmon (*Salmo salar*) y=0.03001x + 0.7027, R^2^ = 0.8118, p < 0.0001

(D) Giant tiger shrimp (*Penaeus monodon*) y=0.02476x + 0.7012, R^2^ = 0.7383, p < 0.0001


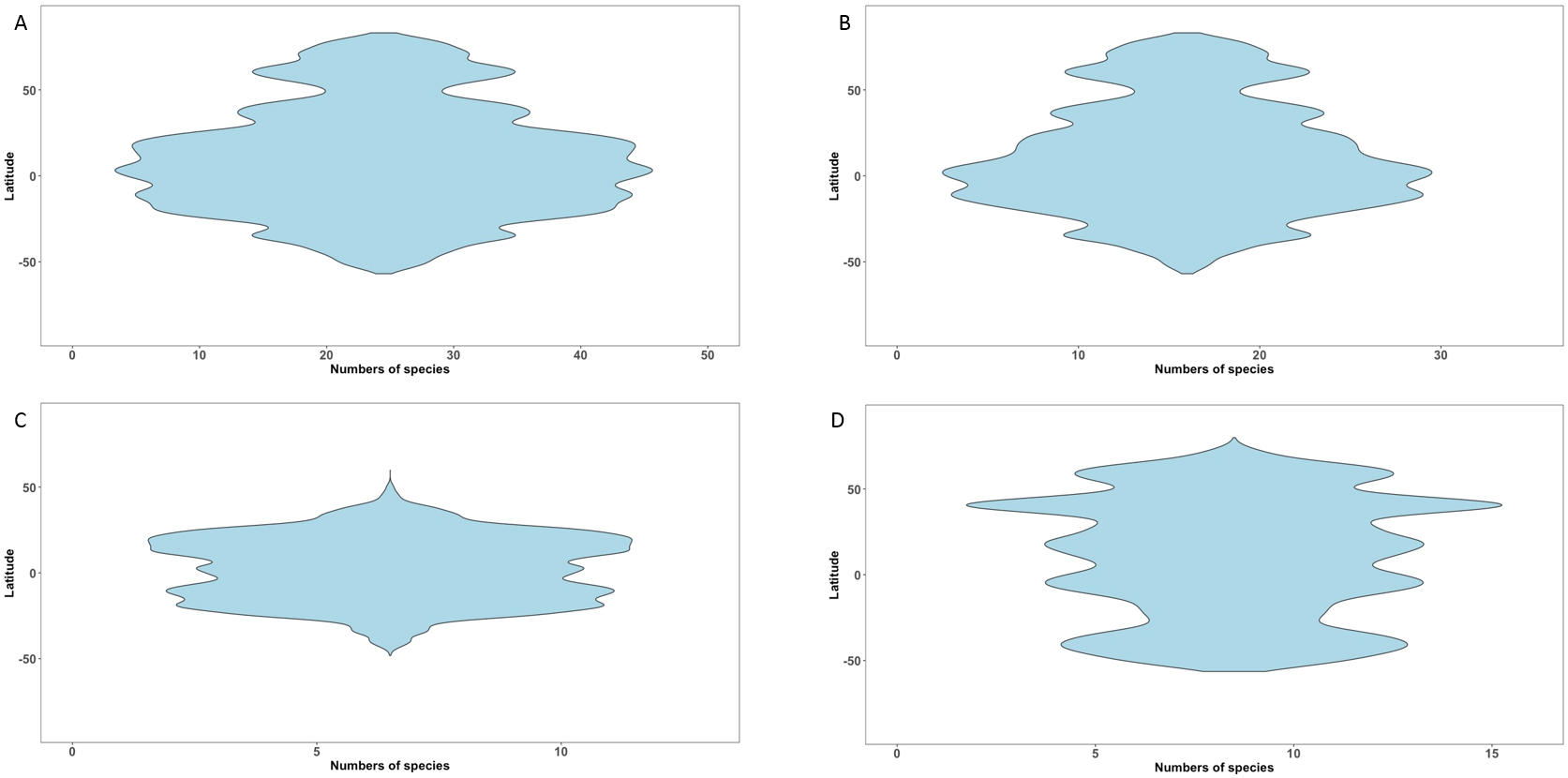


**S4 Fig: Latitudinal predicted species richness** (A) global mariculture species richness (B) Finfish (C) Crustacean (D) Molluscs


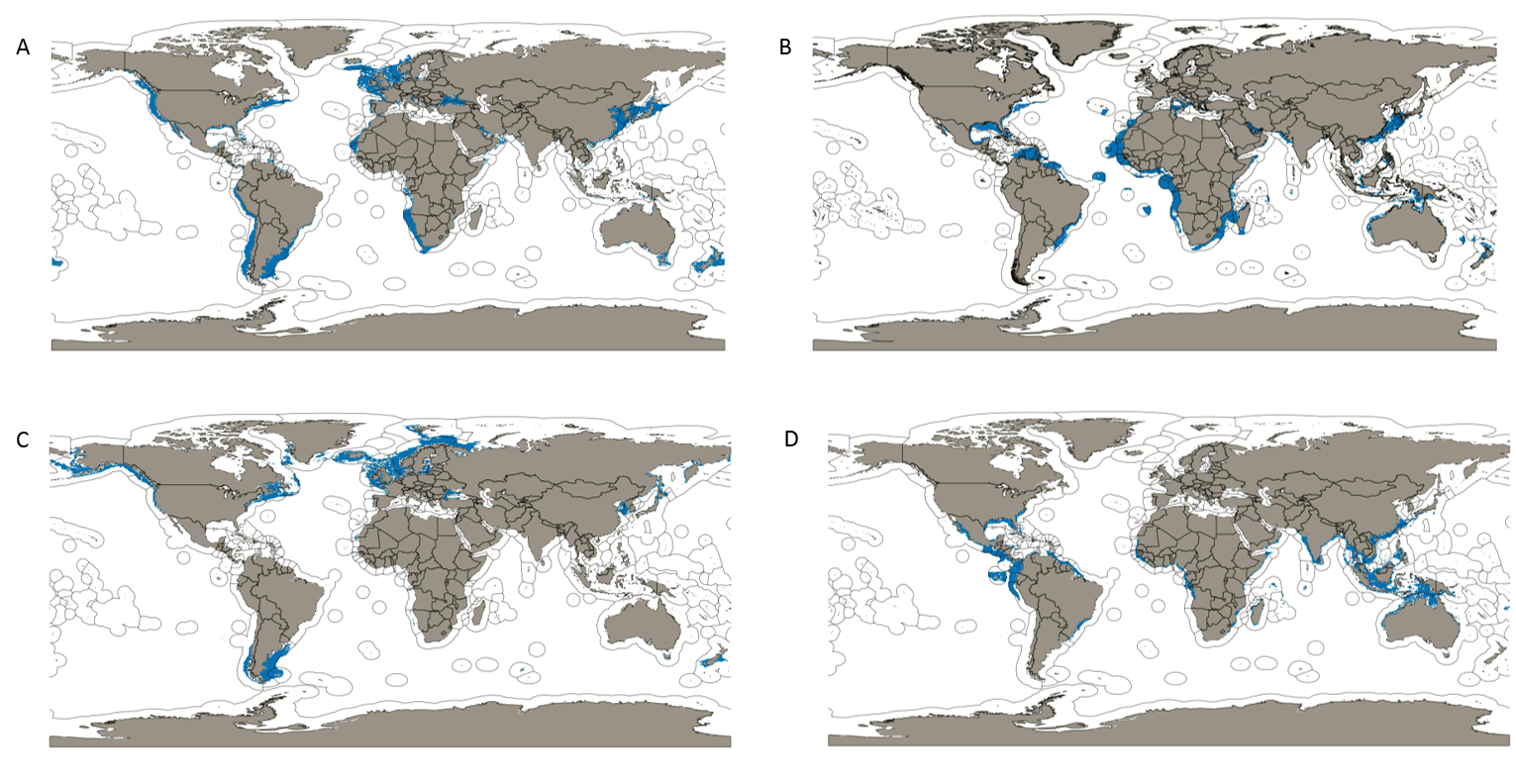


**S5 Fig: Suitable marine areas for farming (A) Pacific cupped oyster (Crassostrea gigas) (B) Cobia (Rachycentron canadum) (C) Atlantic salmon (Salmo salar) (D) Giant tiger shrimp (Penaeus monodon)**

**Reference:**

1. Basille M, Calenge C, Marboutin E, Andersen R, Gaillard JM. Assessing habitat selection using multivariate statistics: Some refinements of the ecological-niche factor analysis. Ecological Modelling. 2008 Feb 24;211(1):233-40.
2. Campbell B, Pauly D. Mariculture: a global analysis of production trends since 1950. Marine Policy. 2013 May 31; 39:94-100.
3. Hutchinson GE. Homage to Santa Rosalia or why are there so many kinds of animals? The American Naturalist. 1959 May 1;93(870):145-59.
4. Jones MC, Cheung WW. Multi-model ensemble projections of climate change effects on global marine biodiversity. ICES Journal of Marine Science. 2014 Oct 10;72(3):741-52.
