# Supplementary figures and images for: Global estimation of areas with suitable environmental conditions for mariculture species
Source: PLoS One. 2018 Jan 19;13(1):e0191086. doi: 10.1371/journal.pone.0191086 (PMC5774971; doi:10.1371/journal.pone.0191086)

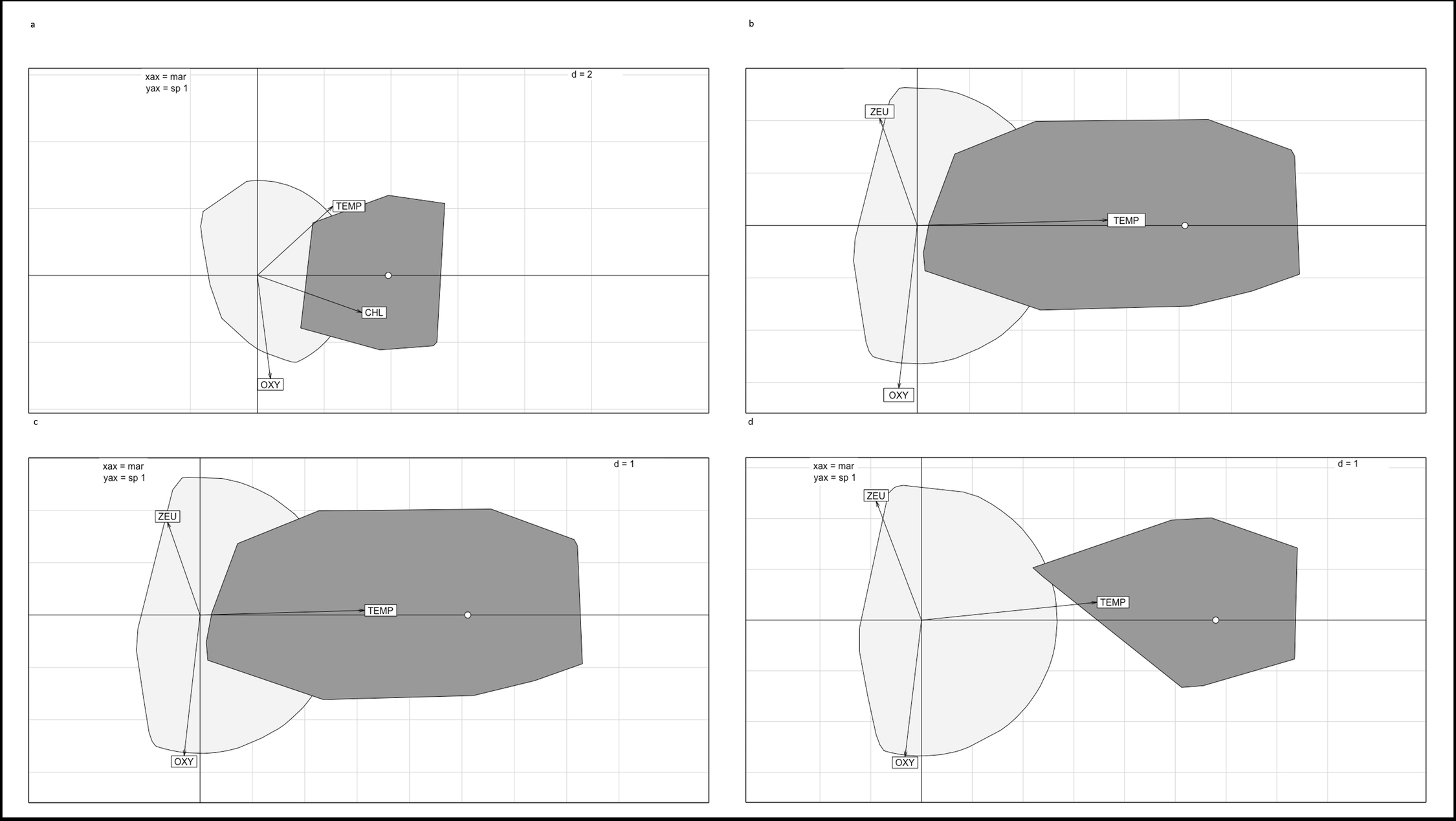

Supplement: S1 Fig — The white dot within the dark area represents the centre of used area while the light area is the available niche. The arrows are projections of oceanic parameters based on mariculture locations of the species (A) Pacific cupped oyster (Crassostrea gigas) (B) Cobia (Rachycentron canadum) (C) Atlantic salmon (Salmo salar) (D) Giant tiger shrimp (Penaeus monodon). (TIF) [file pone.0191086.s002.tif]

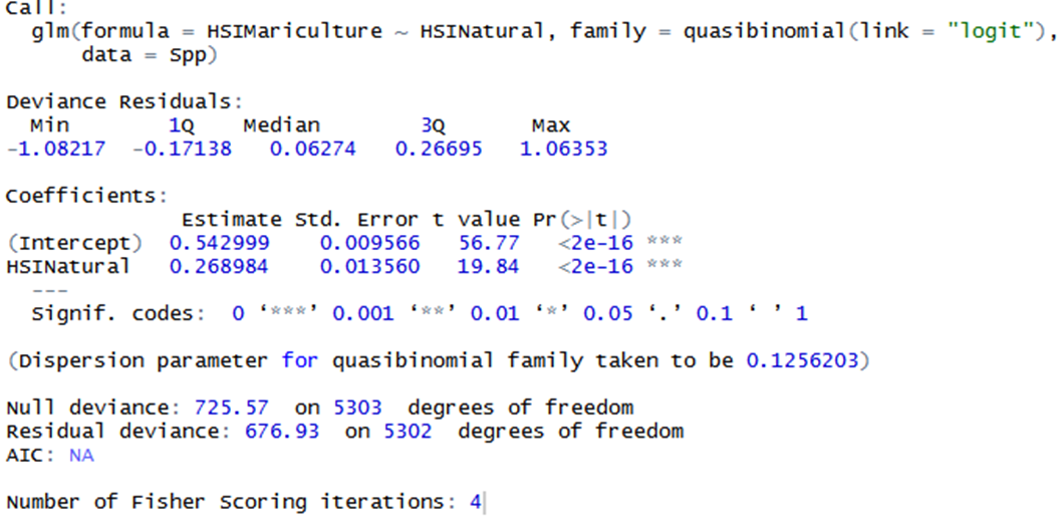

Supplement: S2 Fig — (TIF) [file pone.0191086.s003.tif]

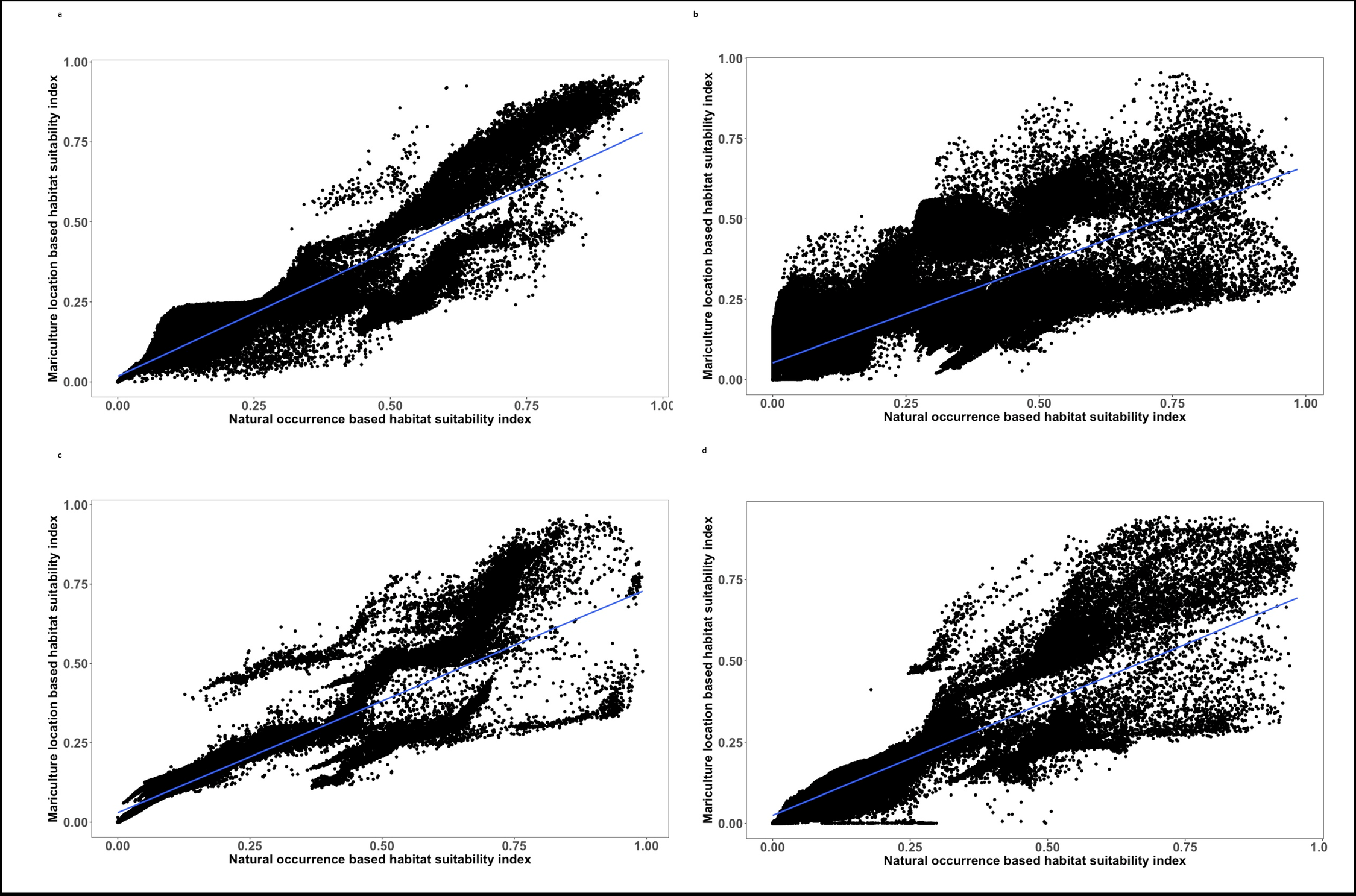

Supplement: S3 Fig — (A) Pacific cupped oyster (Crassostrea gigas) y = 0.01773x + 0.7906, R2 = 0.8607, p < 0.0001. (B) Cobia (Rachycentron canadum) y = 0.04762x + 0.5525, R2 = 0.5951, p < 0.0001. (C) Atlantic salmon (Salmo salar) y = 0.03001x + 0.7027, R2 = 0.8118, p < 0.0001. (D) Giant tiger shrimp (Penaeus monodon) y = 0.02476x + 0.7012, R2 = 0.7383, p < 0.0001. (TIF) [file pone.0191086.s004.tif]

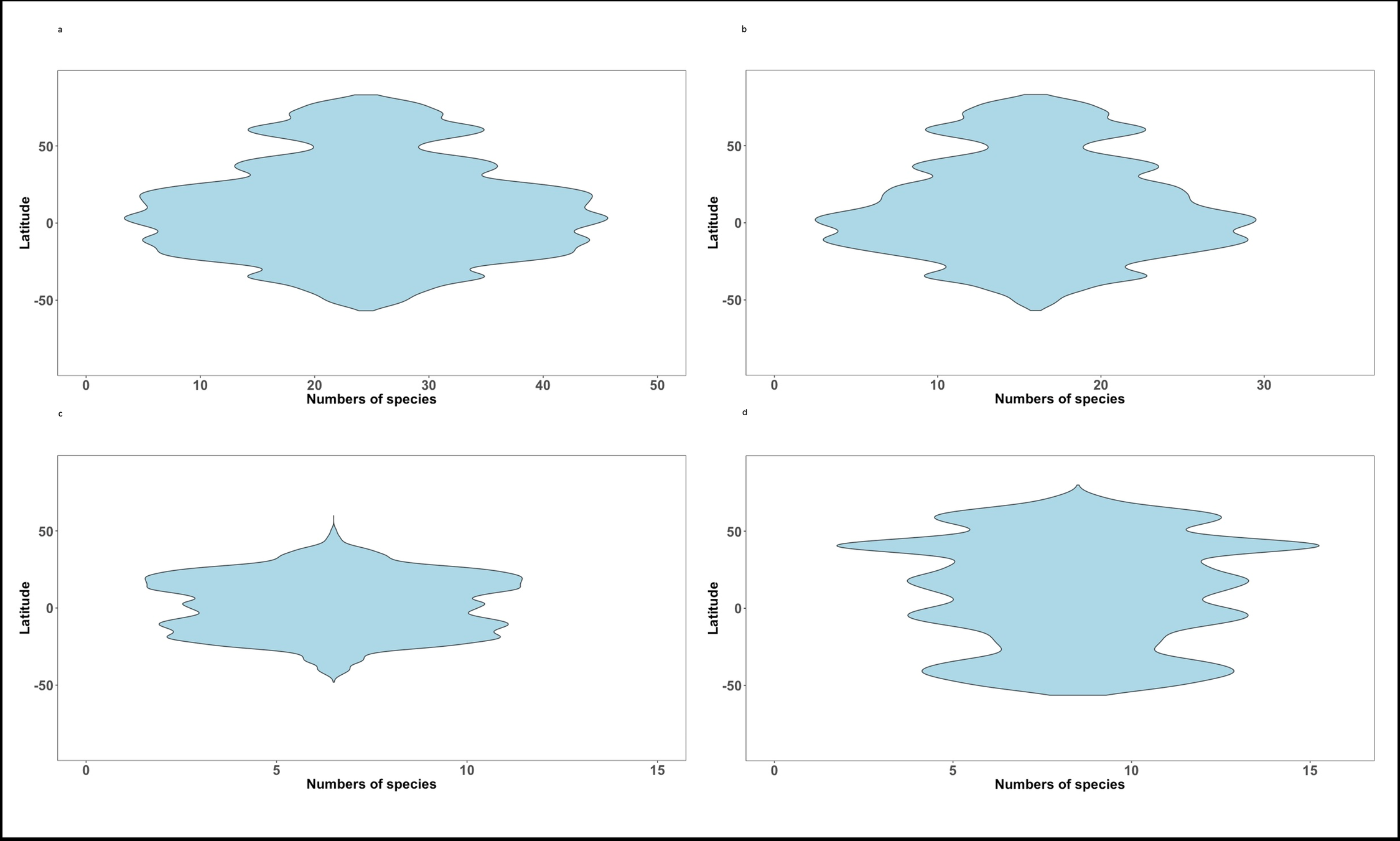

Supplement: S4 Fig — (A) global mariculture species richness (B) Finfish (C) Crustacean (D) Molluscs (TIF) [file pone.0191086.s005.tif]

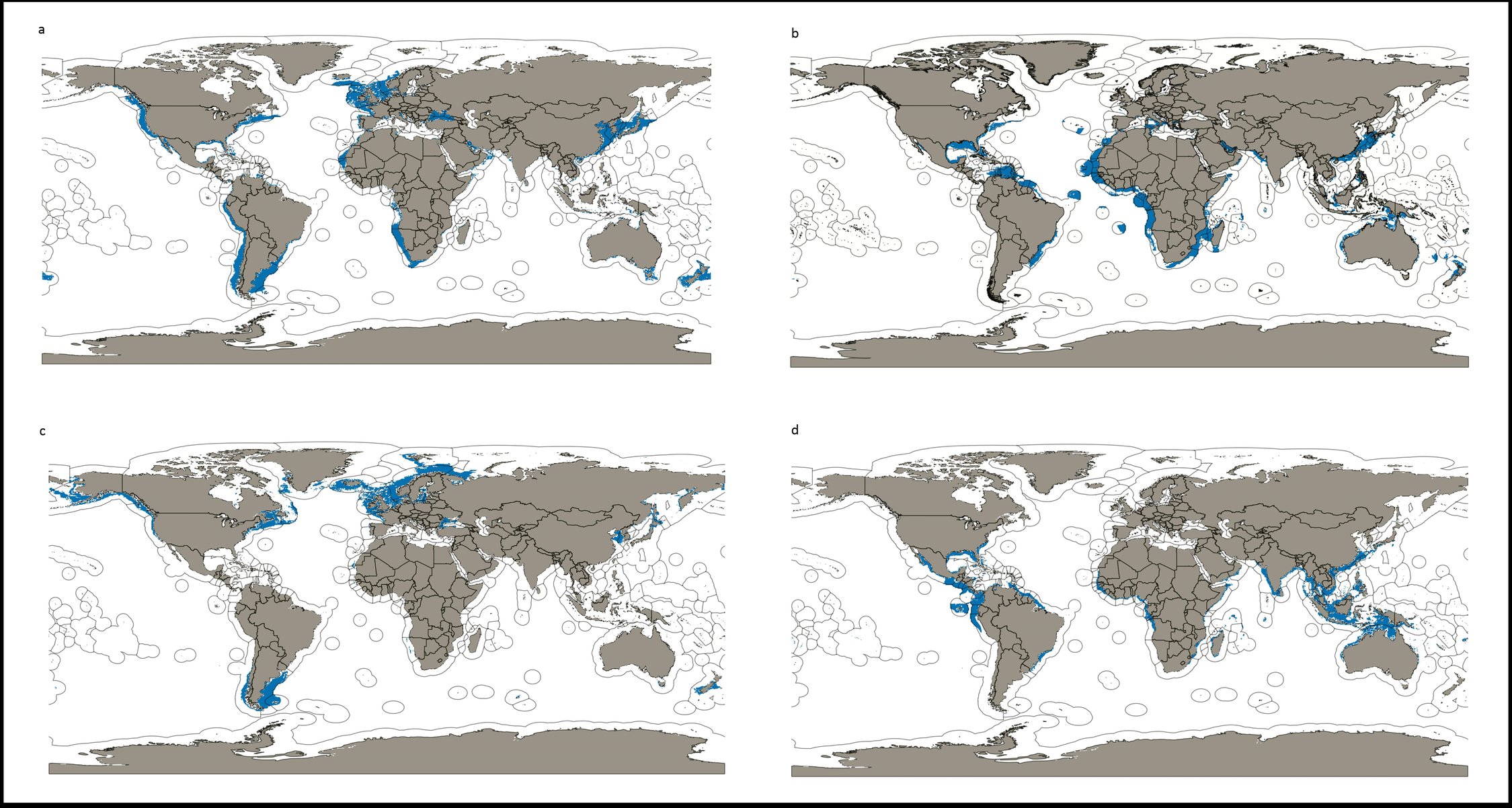

Supplement: S5 Fig — (A) Pacific cupped oyster (Crassostrea gigas) (B) Cobia (Rachycentron canadum) (C) Atlantic salmon (Salmo salar) (D) Giant tiger shrimp (Penaeus monodon). (TIF) [file pone.0191086.s006.tif]
